# Allergenic cross-reactivity between pellitory and mulberry: case report

**DOI:** 10.1186/2045-7022-1-S1-P44

**Published:** 2011-08-12

**Authors:** Laura Losappio, Francesco Contento, Antonio Falco, Cosimo Damiano Cannito

**Affiliations:** 1 Dimiccoli Hospital, Emergency Department, Barletta, Italy

## Background

In Barletta, a city of Puglia in southern Italy, between May and June 2009, at the 'Dimiccoli' Hospital, Emergency Department, 189 people out of 4440 (about 4%) have been found positive to disturbances of allergic type. On May 25th a hot day (max. temperature: 28°C) immediately after a thunderstorm, 15 patients out of 189, rushed to the above mentioned Hospital. Among those 15 patients, there was a 51 years old woman who complained of oculo-rhinitis, cough, chest tightness after being outside on her balcony once it stopped raining. Furthermore she felt face edema and worsening of respiratory symptoms after about 30 minutes from eating few mulberries. Since the clinical outcome was that of "anaphylaxis", the woman was treated with cortisone and antihistamines injection, with successful feedback.

## Methods

The patient went through skin prick tests for inhalants and foods. During the visit she reported a parental medical history of respiratory issues even though no significant history of allergy problems was found.

## Results

The skin prick tests for food allergens and inhalants were positive for pellitory and mulberry. It is worth taking into account that the May 25th there was a high concentration of pellitory pollen (19 granules/m3) and the patient ingested few mulberries.

## Conclusions

This is a case report of allergy to mulberry and to pellitory which confirmed the theory of allergenic cross-reactivity between these two allergens. Furthermore it proved the synergy effect that balanced on the clinical worsening of the patient after inhalation of pellitory pollen and ingestion of mulberry which ended up with the clinical outcome of anaphylaxis.

